# Whole-genome sequences of *Kitasatospora* sp. CMC57 and *Streptomyces* sp. CMC78 isolated from Japanese agricultural soil

**DOI:** 10.1128/mra.00900-24

**Published:** 2025-07-08

**Authors:** Tomoyoshi Hashimoto, Takeshi Shoda, Megumi Ito, Mai Iwamoto-Shizukuda, Daisuke Fukahori, Midori Sakoda, Tomohiro Morohoshi, Masahiro Mitsuboshi, Tomoyasu Nishizawa

**Affiliations:** 1 Tsukuba Research Institute, Katakura & Co-op Agri Corporation, Tsuchiura, Ibaraki, Japan; 2 Graduate School of Agriculture, Ibaraki University 12819, Ami, Ibaraki, Japan; 3 Green-Bio Technology Research Center, Ibaraki University 12819, Ami, Ibaraki, Japan; 4 Graduate School of Regional Development and Creativity, Utsunomiya University 13144, Utsunomiya, Tochigi, Japan; University of Strathclyde, Glasgow, United Kingdom

**Keywords:** actinomycetes, soil microbiology

## Abstract

Herein, we report the nearly complete genome sequences of Actinobacteriota *Kitasatospora* sp. CMC57 and *Streptomyces* sp. CMC78, which were isolated from agricultural soils in Japan.

## ANNOUNCEMENT

Actinomycetes of the genus *Kitasatospora* represent a significant taxonomic group due to their unique combination of characteristics bridging the gap between *Streptomyces* spp. and rare actinomycetes (1, 2). Although considerable interest in actinomycetes is attributable to their reputation as a prolific source of bioactive metabolites, many *Streptomyces* species have been discovered in the rhizospheres of agricultural crops (3, 4). Strains CMC57 and CMC78 were isolated from gray lowland soil (Fluvisols) from Hyogo (35.07 N, 135.25 E) and Ibaraki (35.90 N, 140.13 E) in Japan, using 10-fold serial dilutions in sterile distilled water of each soil (up to 10^6^ dilution) replated on a carboxymethylcellulose agar plate (5) at 15°C for 7 days and at 10°C for 14 days, respectively.

Genomic DNA (gDNA) was extracted from colonies of CMC57 and CMC78 cells grown in R2A (FUJIFILM Wako Pure Chemical Corporation, Japan)—15% agar plate at 30°C for 2 days (CMC57) or 3 days (CMC78) with a DNeasy Power Soil kit (Qiagen, Germany) according to the manufacturer’s protocol. Nanopore sequencing libraries using gDNAs from CMC57 and CMC78, respectively, were prepared using a Native Barcoding Kit (SQK-NBD114.24; Oxford Nanopore Technologies, UK) and sequenced on a PromethION platform (R10.4.1, ONT). Base calling and adapter sequence removal were performed using Guppy v.6.5 with SUP model (6) and Porechop v.0.2.4 (7), respectively. High-quality reads were selected using NanoFilt v.2.7.1 (‘-l 1,000’ and ‘-q 12’) (8). Additionally, 150 bp paired-end libraries using the same gDNA extractions, respectively, were prepared using an MGIEasy PCR-free DNA library prep set (MGI Tech, China) and sequenced on the DNBSEQ-G400 platform (MGI Tech). Adapter-sequence removal and read filtering were performed using Cutadapt v.4.1 (‘--overlap 10’, ‘--minimum-length 51’ and ‘--quality-cutoff 20’) (9) for MGI-reads. CMC57 Nanopore reads were assembled using Flye v.2.9.3 (10) to determine a circular contig and a linear contig, which were subsequently polished by one round of assembly using Medaka v.1.7.2. A circular contig and a linear contig were also obtained for CMC78. The Burrows-Wheeler Aligner v.0.7.17 (11) and Pilon v.1.23 (12) were used with the CMC57-short-read data set shown in Table 1. The same procedure was performed with the CMC78 read. The genome sequences were annotated using DFAST v.1.6.0 (13), and genome completeness was identified using CheckM v.1.2.2 (14) to assess genome quality. The whole genome was identified phylogenomically using JSpeciesWS v.4.2.1 (15). Default parameters were used for all software unless otherwise indicated.

**TABLE 1:** Genome sequences and genome features of *Kitasatospora* sp. CMC57 and *Streptomyces* sp. CMC78

Strain	*Kitasatospora* sp. CMC57				*Streptomyces* sp. CMC78			
Sequencer	Nanopore	MGISEQ			Nanopore	MGISEQ		
Total length (bp)	1,571,758,694	1,939,678,200			1,757,940,634	2,340,915,000		
No. of raw reads	174,414				237,620			
No. of reads post-filtering	90,882				149,055			
No. of raw read pairs		6,465,594				7,803,050		
No. of read pairs post-filtering		6,465,587				7,803,025		
Ave. length (bp)	10,086				7,786			
N50 value	15,487				11,171			
Genome features			Contig1	Contig2			Contig1	Contig2
Alias			chromosome	plasmid			chromosome	plasmid
Form			linear	circular			linear	circular
Genome size (bp)			7,067,669	97,860			8,198,216	9,621
N50 value			7,067,669				8,198,216	
GC content (%)			71.7	70.8			71.7	71.6
No. of CDSs			6,383	155			7,380	12
No. of rRNAs			27				18	
No. of tRNAs			108	1			78	
No. of tmRNAs			1				1	
Completeness (%)			98.86				99.91	
DRA/DDBJ accession no.	DRR582519	DRR582520	AP035881	AP035882	DRR582521	DRR582522	AP035884	AP035885

Table 1 shows the genome features of strains CMC57 and CMC78. The linear chromosome ends of CMC57 and CMC78 were not determined, respectively. To confirm the taxonomic placement using the genome sequence of the most closely related species, whole-genome average nucleotide identity was examined using whole-genome sequences of *K. gansuensis* DSM 44786^T^ (NCBI accession number GCA_014203705.1) and draft genome sequences of *S. griseus* NRRL ISP-5322 (GCF_002154345.1) had 92.61% and 98.34% sequence identities, respectively, with strains CMC57 and CMC78.

## Data Availability

The genome sequences of strains CMC57 and CMC78 were deposited in DDBJ/ENA/NCBI under the accession numbers and SRA numbers listed in Table 1, respectively.
